# The fundamentals of cultural adaptation: implications for human adaptation

**DOI:** 10.1038/s41598-020-70475-3

**Published:** 2020-08-31

**Authors:** Laurel Fogarty, Anne Kandler

**Affiliations:** Theory in Cultural Evolution Lab, Department of Human Behavior, Ecology and Culture, Max Planck Institute for Evolutionary Anthropology, Leipzig, Germany

**Keywords:** Evolution, Cultural evolution

## Abstract

The process of human adaptation to novel environments is a uniquely complex interplay between cultural and genetic changes. However, mechanistically, we understand little about these processes. To begin to untangle these threads of human adaptation we use mathematical models to describe and investigate cultural selective sweeps. We show that cultural sweeps differ in important ways from the genetic equivalents. The models show that the dynamics of cultural selective sweeps and, consequently, their differences from genetic sweeps depend critically on cultural transmission mechanisms. Further, we consider the effect of processes unique to culture such as foresight and innovations in response to an environmental change on adaptation. Finally we show that a ‘cultural evolutionary rescue’, or the survival of an endangered population by means of cultural adaptation, is possible. We suggest that culture might make a true, genetic, evolutionary rescue plausible for human populations.

## Introduction

Genetically, a population can adapt to a new niche or a novel environment in two ways, either by relying on existing genetic variation, or through the appearance of beneficial new mutations^1,2^. In the latter case the rate of adaptation is limited by the rate of genetic mutation, and in the former the adaptive process is constrained by the variance in a relevant trait, and influenced by selection and drift prior to entering the new environment^1–3^. This difference has important implications for estimates of past and future rates of genetic evolution and the discernible signatures of selective sweeps in each case^2^.

The potential for a rapid rate of cultural evolution compared to genetic change raises an important possibility: in humans, genetic adaptation to new environments or genetic responses to environmental shifts may be preceded by much more rapid cultural adaptation^4^. Indeed, the ability of modern humans to adapt to novel environments is often attributed to our uniquely well-developed ability to rapidly amass large adaptive cultural repertoires^5,6^. However, while the field of cultural evolution has provided deep insights into the processes of cultural transmission^5,7^, we still understand little about how cultural adaptation to novel environments might proceed, what if any, evidence of past selection might be found in cultural data, and how this might interact with genetic adaptation or interfere with genetic signatures of selective events.

For example, in analogy with the genetic case described above, does *cultural* adaptation generally occur from existing (standing) variation or from an innovation-limited process? It may be reasonable to assume that the process of cultural innovation proceeds more rapidly than genetic mutation but what does this mean for our understanding of adaptation and the signatures of adaptation? Understanding these dynamics demands close examination of the way in which we typically model innovation in cultural evolutionary systems (see Ref.^8^). Often, the rate of innovation in cultural evolutionary models is formulated as a random process analogous to genetic mutation. In many cases, this is a good approximation to the way in which humans innovate^9,10^. However, in addressing questions about cultural adaptation, this might not be the case for a number of reasons.

For example, the adaptive value of a cultural variant innovated prior to an environmental change might, on average, be lower than a variant innovated in direct response to that change. Such differences may create trade-offs between standing variation and de novo innovations in cultural systems that do not exist in their genetic analogues. In turn, these effects could change the balance of probability of adaptation from standing variation or from novel innovation. The speed and timing of genetic adaptation can be influenced by the rate of mutation or by selection on standing variation in the time preceding an environmental change. In the case of culture, as in genetics, adaptation might be affected by prior selection on cultural variants and by the rate of random innovation. However, it might also be affected by additional processes unique to culture. For example, the effectiveness and relative importance of the kind of ‘directed innovation’ discussed above as well as the dominant modes of cultural transmission that might cause a population to conserve or eliminate standing cultural variation in a way that differs considerably from neutrality or a case of direct natural selection.

Understanding the process of adaptation has deep implications for our understanding of population survival in the face of changing environments. This is often a concern for conservation biologists interested in mitigating the effects of human activity on plant and animal populations^11,12^. However, in the case of human evolution itself, an understanding of adaptation to drastically changing environments might also help us to elucidate the history of our species. Why, for example, is the human species so good at moving, and adapting, to such a variety of extremely diverse habitats when other great ape species are not? Here we suggest that beneficial cultural traits act in two ways to help human populations survive drastic environmental shifts. First, the presence of the cultural trait can itself compensate for a mismatch with the environment and mitigate the consequences of genetic maladaptation. And second, the presence of even a weakly beneficial cultural trait might allow a population to survive longer with a slowly declining population, increasing the probability of a true evolutionary rescue—a possibility usually discounted in species with long generation times such as ours.

Here we describe models of cultural adaptation that examine the probability of adaptation from standing cultural variation compared to that for adaptation from novel innovation. We examine the implications for human evolution in general and discuss the importance of including gene-culture co-evolution in models of human adaptation in particular. Finally, we discuss what role cultural adaptation might play in evolutionary rescue and the preservation and persistence of human populations faced with local extinction.

## Fixation probability from standing cultural variation or de novo innovation

In the following we consider a cultural trait with two alternative variants, *a* and *A*, where *A* is ancestral and *a* represents a novel innovation. We calculate probability of fixation for the novel innovation in two different adaptation scenarios: adaptation from de novo innovation and adaptation from standing variation. Adaptation from de novo innovation occurs where the variant *a* arose in a single individual (i.e. with frequency 1/*N*) after an environmental shift and therefore corresponds to a situation where a beneficial innovation is found once the environment has changed. Adaptation from standing variation occurs where the novel variant *a* was present in the population at some frequency at the time of the environmental shift. These correspond to a situation where where a cultural response to a new environment is found after the environment has changed and one where the population’s existing cultural repertoire already contains a response to the changed environmental conditions, respectively.

### Model set-up

The population is finite containing *N* individuals each possessing one variant of the cultural trait, either *a* or *A*. In each time step a new individual arises and adopts variant *a* or *A*, before replacing another randomly selected individual, who dies^13^. Initially, neither variant provides an adaptive benefit and both are transmitted from a randomly chosen role-model to a newborn with a probability equal to its frequency. In other words, the variants evolve neutrally, through unbiased transmission. At time $T_0$ an environmental shift occurs. After this, variant *a* provides an adaptive benefit *f* and *A* provides a benefit *g*, with $f>g$. Now, the new individual chooses a role model with a probability weighted by *g* and *f* and adopts its role-model’s cultural variant. Thus, the variants *a* and *A* now evolve through payoff-biased transmission^5^ and *f* can be interpreted as the cultural transmission advantage of variant *a*. In both transmission regimes, biased and unbiased, the time evolution of the number of variants of type *a* present in the population at time *t* can be modelled as a Markov process $\{X_t : t \ge 0\}$ with values in the set $\{0,1,\ldots ,N\}$. The process $N-X_t$ describes the evolution of trait *A*.

### The probability of fixation from de novo innovation

We calculate the probability that the novel variant *a* will fix given that it arose after the environmental change at frequency 1/*N*. The transition probabilities for the Markov process $X_t$ in this case are given by

$$\begin{aligned} p_{i,i-1}= & {} \frac{g(N-i)}{fi+g(N-i)} \frac{i}{N}=\beta _i\\ p_{i,i+1}= & {} \frac{fi}{fi+g(N-i)}\frac{N-i}{N}=\alpha _i\\ p_{i,i}= & {} 1-p_{i,i+1}-p_{i,i-1}=1-\alpha _i-\beta _i, \quad i=1,\ldots ,N-1 \end{aligned}$$

where $p_{i,\cdot }$ describes the probability that the absolute frequency of variant *a* in the population changes from *i* to $i-1,i$ or $i+1$ in one time step. Further, *f* quantifies the benefit of variant *a* after the environmental change and *g* the benefit of variant *A* (assumed to be 1 in the following). It can be shown (see Supplementary Section S1 in the supplementary material for a detailed derivation) that the probability of fixation from a de novo innovation with adaptive benefit *f* is given by

1 $$\begin{aligned} \pi _\text {DN} = \pi _1=\frac{1}{1+\sum \nolimits _{l=1}^{N-1}\prod \nolimits _{k=1}^{l}\frac{\beta _k}{\alpha _k}}. \end{aligned}$$

### The probability of fixation from standing variation

Next, we assume that the innovation of variant *a* occurred some time before the environmental change and unbiased transmission has caused it to reach frequency *j*/*N*, with $j=1,\ldots ,N-1$ at $T_0$. We condition on the existence of a variant *a* that has not yet reached fixation in the population and consequently, the probability of fixation of *a* after the environmental shift will depend not just on the benefit of *a* but also on the expected frequency of *a* at $T_0$. To calculate the fixation probability we first calculate the probability that variant *a* has frequency *j*/*N* under unbiased transmission and multiply this by the probability of fixation from frequency *j*.

In the case of unbiased transmission, the transition probabilities of the Markov process $X_t$ are given by

$$\begin{aligned} p_{i,i-1}=p_{i,i+1}= & {} \frac{i(N-i)}{N^2}=a_i,\\ p_{i,i}= & {} \frac{i^2+(N-i)^2}{N^2}=1-2a_i,\quad i=1,\ldots ,N-1. \end{aligned}$$

The probability that variant *a* has reached frequency *j*/*N* is given by $\frac{t_{1j}}{t_1}$ where $t_{1j}$ denotes the mean time that the Markov process $X_t$ with the initial condition $X_0=1$ was in state *j* and $t_1$ is the mean time that variant *a* exists before absorption into either state 0 or *N*. It holds (see Supplementary Section S2 in the supplementary material for a detailed derivation) that

2 $$\begin{aligned} t_{1j}=\frac{N}{j} \end{aligned}$$

and

3 $$\begin{aligned} t_{1}=N\left( 1+\sum \limits _{k=2}^{N-1}\frac{1}{k}\right) \end{aligned}$$

which leads to

$$\begin{aligned} \frac{t_{1j}}{t_1} = \frac{1}{j\left( 1+\sum \nolimits _{k=2}^{N-1}\frac{1}{k}\right) }. \end{aligned}$$

Finally, we generalise the expression for the fixation probability (1) from a starting frequency of 1 to a general starting frequency of *j* using the fact that

$$\begin{aligned} \pi _j=\pi _1+\pi _1\sum _{l=1}^{j-1}\prod \limits _{k=1}^l\frac{\beta _k}{\alpha _k} \end{aligned}$$

and obtain

$$\begin{aligned} \pi _j=\frac{1+\sum _{l=1}^{j-1}\prod _{k=1}^{l}\frac{\beta _k}{\alpha _k}}{1+\sum _{l=1}^{N-1}\prod _{k=1}^{l}\frac{\beta _k}{\alpha _k}}. \end{aligned}$$

So the probability of a fixation from standing cultural variation, at the time of an environmental change, i.e. from a variant with frequency *j*/*N* at $T_0$ and an adaptive benefit *f*, is given by

4 $$\begin{aligned} \pi _\text {SV}=\sum _{j=1}^{N-1} \frac{t_{1j}}{t_{1}}\cdot \pi _j. \end{aligned}$$

**Figure 1:**
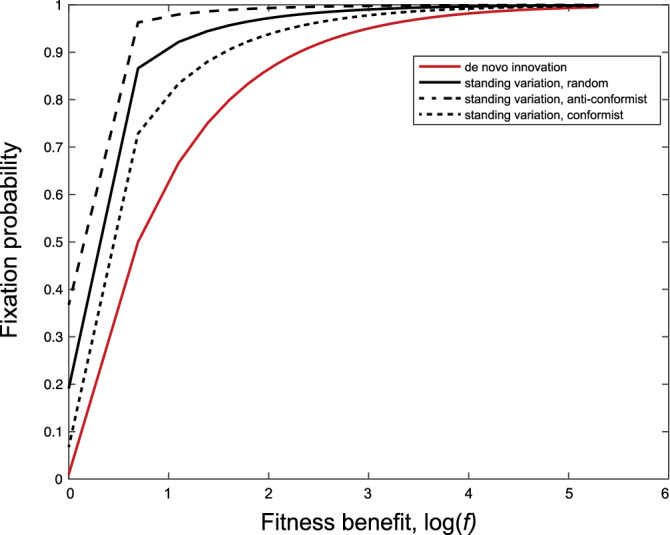
(**A**) The probability of a cultural selective sweep from standing variation generated by unbiased transmission (black solid line), conformity with $\theta =0.5$ (dashed-dotted line), anti-conformity with $\theta =-0.5$ (dashed line) or from de novo innovation (red line) after an environmental change.

Summarising, the probabilities $\pi _\text {DN}$ (1) and $\pi _\text {SV}$ (4) express how likely trait *a* with benefit *f* goes to fixation when it is a *de novo* innovation or part of the existing cultural repertoire of the population, respectively. Figure 1 illustrates those probabilities for various values of *f*. The fixation probability is lowest if the adaptive trait is a de novo innovation, i.e. invented at $T_0$ with frequency 1/*N* for all values of *f* (compare red line for de novo innovation and black line for standing variation). This is an intuitive finding as standing variation can result in situations where the frequency of the adaptive variant *a* is larger than 1/*N* at the time of the environmental shift what in turn leads to a higher fixation probability. Before we discuss some implications of these results for the theory of cultural adaptation, we consider the influence of transmission processes other than unbiased transmission on the fixation probability.

### The probability of fixation from standing variation under alternative transmission mechanisms

An important difference between genetic and cultural evolution is the large number of different ways in which information can be passed on from one generation to the next in a cultural context^14^. Cultural transmission processes affect how cultural traits are maintained or lost in a population^4^. As a result, it is possible that the probability of a sweep to fixation might depend on the cultural transmission processes on which a population relies before an environmental change. To quantify the effects of alternative transmission processes, we need to generalise Eq. (4) to allow for the general transition probabilities $p_{i,i-1}=\beta _i, p_{i,i+1}=\alpha _i, \text {and } p_{i,i}=1-\alpha _i-\beta _i$ We only consider transmission processes whose temporal dynamic is Markovian. In doing so (see Supplementary Section S3 in the supplementary material for a detailed derivation) we obtain for the mean time to absorption, $t_1$, and mean time spent at a given frequency, $t_{1j}$

5 $$\begin{aligned} t_{1j}=\frac{\frac{(N-j)}{\alpha _j}\prod \nolimits _{k=j+1}^{N -1}\frac{\beta _k}{\alpha _k}}{N\prod \nolimits _{l=1}^{N-1}\frac{\beta _l}{\alpha _l}}. \end{aligned}$$

and

6 $$\begin{aligned} t_{1}=\sum _{j=1}^{N-1}t_{1,j}. \end{aligned}$$

Substituting these expressions in Eq. (4) provides us with the fixation probability from standing variation assuming an arbitrary cultural transmission process defined by the transition probabilities $\alpha _i$ and $\beta _i$. In other words, we can derive the fixation probabilities for any cultural transmission process for which we can formulate the transition probabilities $\alpha _i$ and $\beta _i$ of the corresponding Markov process. We note that the population still applies payoff-biased transmission after the environmental shift.

**Figure 2:**
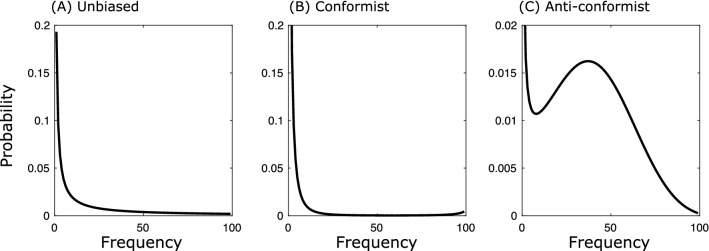
Probability that variant *a* has frequency *j* shown on the x-axis at $T_0$ under (**A**) unbiased transmission, (**B**) conformity with $\theta =0.05$, and (**C**) anti-conformity with $\theta =-0.05$.

To illustrate the potential effect of cultural transmission processes on the probability of a sweep to fixation, we assume that transmission before $T_0$ is governed by a frequency-dependent bias, i.e. the tendency to disproportionately copy either common variants (conformity) or rare variants (anti-conformity)^5^. In this case the transition probabilities are given by

7 $$\begin{aligned} p_{i,i+1}= & {} \frac{(i/N)^{(1+\theta )}}{(i/N)^{(1+\theta )}+(1-(i/N))^{(1+\theta )}}\frac{N-i}{N}=\alpha _i,\nonumber \\ p_{i,i-1}= & {} \frac{((N-i)/N)^{(1+\theta )}}{(i/N)^{(1+\theta )}+(1-(i/N))^{(1+\theta )}}\frac{i}{N}=\beta _i,\nonumber \\ p_{i,i}= & {} 1-\beta _i-\alpha _i. \end{aligned}$$

where $\theta >0$ models conformity and $\theta <0$ anti-conformity. Calculating the fixation probability (4) using Eqs. (5)–(7) allows us to compare the probability of a cultural sweep under different transmission processes prior to $T_0$. Figure 1 shows that, compared to unbiased transmission (see black solid line), conformity (see short dashed line) and anti-conformity (see long dashed line) show higher fixation probabilities for all values of *f*. This is because conformity reduces the probability that variant *a* has high frequency at $T_0$, while anti-conformity increases the probability that a variant is maintained at an intermediate frequency. This is shown in Fig. 2, which shows the frequency distribution of a variant for the three transmission processes considered above).

As a side note, knowing the ratio $t_{1j}/t_1$ allows us to derive a kind of ‘trait frequency spectrum’ for an infinite sites Moran model^15^ under the transmission process defined by $\alpha _i$ and $\beta _i$. The number of variants with frequency *j* in the population, denoted by $S_{N,j}$, is given by

$$\begin{aligned} S_{N,j}=\frac{t_{1j}}{t_1}S_N=t_{1j}\mu \end{aligned}$$

where $S_N$ represents the average number of cultural variants expected to be present in the population at some timestep *t* and $\mu$ the *per capita* innovation rate. (for more detail see Supplementary Section S4 in the supplementary material).

### Implications for the theory of cultural adaptation

The results so far have shown that if the standing cultural variation in a population contains a variant that becomes adaptive after an environmental change, then the probability of a sweep to fixation is higher compared to a situation where an adaptive variant with the same level of benefit is invented after an environmental shift. This result is intuitive—standing variation is likely to produce variants with frequencies larger than 1/*N* at $T_0$ and these variants are at an advantage compared with those at lower frequency. These results suggest it is plausible that under some circumstances populations need not, and indeed should not, rely on inventing novel traits in novel environmental conditions if they possess adaptive standing variation. Naturally this raises further questions such as ‘under what circumstances do populations possess adaptive standing cultural variation, and what mechanisms produce and maintain it?’. In other words, exploring the mechanisms that can generate standing variation containing an adaptive variant after an arbitrary environmental change is of great interest. An extensive analysis of these questions might require an *n* variant model to allow for the accumulation of cultural diversity and this is a subject of future research.

In the next section, as above, we explore in the two variant model a simple mechanism capable of generating and maintaining standing variation: foresight. We note that there are a number of candidate mechanisms capable of maintaining cultural variation such as frequent environmental changes, accurate copying of vast bodies of cultural knowledge, relatively accurate foresight, or high innovation rates. Here, we investigate just one simple mechanism.

## Foresight vs. innovation: uniquely cultural tradeoffs in the probability of selective sweeps

So far we have assumed that standing variation, or the cultural repertoire, is maintained by unbiased or frequency-biased transmission. In other words, the temporal dynamic of the variant *a* before $T_0$ is driven by their frequency and not their intrinsic properties. There may be good reason to believe that the adaptive benefit of variants derived under such neutral conditions might be lower than variants invented in direct response to new environmental conditions. This means that a trade off may exist in a cultural system that does not exist in its genetic analogue: the adaptive value of cultural variants innovated under different circumstances might, on average, differ, with variants innovated in direct response to an environmental challenge having higher average adaptive value compared to those that are innovated prior to that challenge. However, this ‘benefit disadvantage’ might be compensated for by the frequency advantage of standing variation.

Of course, standing variation doesn’t have to be fully blind to future environmental changes and the cultural repertoire can be generated by processes other than random innovation. Here we allow for foresight, which might enable populations to invent or maintain variants in their cultural memory which could be of use after an environmental change. However, we note that the existence and implications of foresight in cultural innovation have been discussed at length by Mesoudi^10^ who argued convincingly that ‘foresight’ should not be confused with a sort of supernatural clairvoyance. Therefore, we do not imply that the adaptive value of a variant innovated or maintained with foresight is always positive, nor that the generation of innovations is not random, only that the underlying distribution of fitness effects might be skewed by our cognitive abilities.

In the following we explore circumstances under which adaptation from de novo innovation is more likely, as a result of an increase in benefit associated with innovation in response to a challenge. We assume that humans can (i) exercise some foresight to produce a cultural variant *a* with mean benefit $f_{SV}$ before the environmental change and (ii) innovate a cultural variant *a* in direct response to the changed environment with benefit $f_{DN}$.

**Figure 3:**
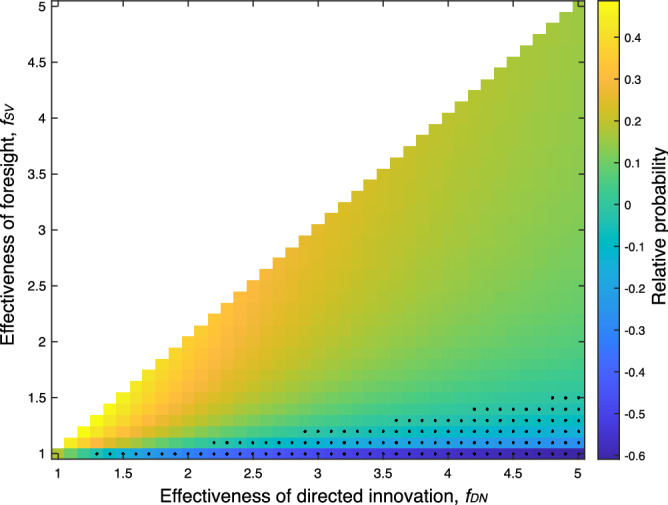
The difference in probability of a sweep from standing variation and a novel innovation with different strengths of ability for foresight (*y* axis) and directed innovation (*x* axis). We assume that foresight cannot perform better than directed innovation and so consider the bottom diagonal only.

Figure 3 shows the difference in fixation probability

$$\begin{aligned} \Delta \pi = \pi _\text {SV}(f_\text {SV}) - \pi _\text {DN}(f_\text {DN}) \end{aligned}$$

with $\pi _\text {SV}(f_\text {SV})$ given by Eq. (4) and $\pi _\text {DN}(f_\text {DN})$ by Eq. (1) for different values of $f_\text {SV}$ and $f_\text {DN}$.

Directed innovations can only compensate for their frequency disadvantage if the level of benefit generated by standing variation is relatively small (see black dots in Fig. 3 which indicate the values of $f_\text {DN}$ and $f_\text {SV}$ leading to $\pi _\text {DN}(f_\text {DN})>\pi _\text {SV}(f_\text {SV})$). This implies that adaptation from standing variation may prove more efficient than adaptation from de novo innovation over a wide parameter range and highlights the importance of understanding the level of adaptivness that can be generated in standing cultural variation. Is it realistic to assume that standing variation can generate cultural variants that provide a high level of benefit after an arbitrary environmental change? Or is it more likely that standing cultural variation proves to be only marginally adaptive by producing variants with a very small benefit? Is variation maintained in the standing cultural variation likely to be maladaptive?

## Evolutionary rescue in humans

Above, we assumed that the level of cultural adaptation to changed environmental conditions has no influence on the survival of the population or on population demography more generally, since, by definition, the population size is constant at *N* even after an environmental change. However, under this assumption, as we have seen in Fig. 1, the probability of a cultural selective sweep is rarely 1, even when the innovation provides a very high benefit compared to the ancestral variant of the trait. This means that the success of a population faced with a novel environment is not guaranteed and one may ask ‘what are the consequences of a failure to adapt?’. One clear answer may be population extinction^16^.

To explore the interplay between cultural adaptation and extinction or extirpation risk we describe an extension to our model drawing on work on “evolutionary rescue”   where a species or population adapts to environmental change sufficiently rapidly to avoid extinction with no inward migration with or without interbreeding (distinct processes known as genetic or demographic rescue respectively)^11,17–22^.

Although it is not widely believed that organisms with large body size and long generation times are likely to undergo evolutionary rescue and remain, instead, the most vulnerable to extinction in changed environments^11,12^, we contend that humans are an exception. In human populations genetic adaptation may be preceded by much more rapid cultural adaptation, as was the case in the smaller and faster breeding field cricket whose evolutionary rescue was accompanied by a facilitating behavioural adaptation^23^. In the case of humans, behavioural and cultural changes may be even more crucial. For example, extensive use of warm weather clothing is thought to have emerged as humans migrated away from the warmer climates in Africa and into Europe^24^ some 500–600 kya^25^. However, at least some important genetic adaptations to the cold in European populations did not reach high frequencies before 3–8 kya^26^. This suggests the possibility that human populations may first survive extreme changes in their environments through behavioural and cultural adaptations, which may be followed by adaptive genetic changes. This might also imply that if cultural adaptation to a novel environment does *not* succeed or does not proceed with sufficient speed, the population will face extinction^16^. Consequently, in cases where cultural adaptation prevents or delays population extirpation, it is crucial to understand cultural adaptation and the dynamics of what we might call ‘cultural rescue’ as distinct from evolutionary, genetic, and demographic rescues^22^.

### Model set-up

As before, each individual in a model population is assumed to have one of two variants of a single cultural trait: an ancestral variant, *A*, or a novel innovation, *a*. At time $T_0$ the environment shifts suddenly and catastrophically. Neither of the two variants have an adaptive benefit in terms of transmission or survival before the shift. After the shift the transmission of variant *a* is weighted by *f* and that of variant *A*, by *g* (with $f>g$). Additionally, both variants affect an individual’s survival probability differently when the environment has changed. We assume that a cultural response to challenging environmental conditions may be more likely to prevent death rather than increase fertility (taking, again, the example of cold weather clothing above). To model this link between cultural adaptation and survival we relax the assumption of constant population size and define the following the birth-death process. Each individual has a per-capita death rate depending on the adopted cultural variant

8 $$\begin{aligned} p_{\text {death},a,t}= & {} \frac{1}{N_t}(1-q)\nonumber \\ p_{\text {death},A,t}= & {} \frac{1}{N_t}(1+r)\quad \text {with}\ q,r<1. \end{aligned}$$

The variable $N_t$ describes the population size at time *t*, *q* the survival benefit of the novel innovation *a*, *r* the increase in the probability of death of an individual with the ancestral variant *A* owing to its mismatch with a new environment. The value of *r* also indicates the initial level of maladaptation, a crucial parameter in determining the probability of population survival^11^.

In each time step individuals die according to their death rate (8) and one naive individual is added to the population who adopts the variant from a chosen role model. This choice is governed by the probabilities

$$\begin{aligned} p_\text {reproduction,a,t}= & {} \frac{fi_t}{fi_t+g(N_t-i_t)}\\ p_\text {reproduction,A,t}= & {} \frac{g(N_t-i_t)}{fi_t+g(N_t-i_t)} \end{aligned}$$

with $f=q=1$ before $T_0$ and $f>g$ after $T_0$. The variable $i_t$ describes the number of individuals having adopted variant *a* at time *t* in the population. In the following we focus on adaptation from *de novo* innovation, i.e. we assume $T_0=0$ and $i_0=1$.

### Characteristics of cultural rescue

Our model implies that the average change in population size in each time step is given by $(i_t/N_t)(q+r)-r$. Consequently we observe, on average, an increase in population size if $r/(q-r)<i_t/N_t$ and a decrease for $r/(q-r)>i_t/N_t$. In other words, the spread of variant *a*, determines the fate of the population. If the fraction of the population with *a* increases fast enough, then the cultural adaptation successfully counteracts the increased mortality caused by the mismatch between the environment and the ancestral variant, *A*. As we assume payoff-biased transmission after the environmental shift at $T_0$ the spread behaviour of variant *a* is determined by the difference between *f* and *g*: the larger the difference, the faster the spread and the more likely and earlier the rescue.

In general, we can formulate the recursion equations describing the time course of the average population size, ${\mathbf {E}}\{N_{t+1}|N_t,i_t\}$, and the average number of individuals with variant *a*, ${\mathbf {E}}\{i_{t+1}|N_t,i_t\}$,

9 $$\begin{aligned} {\mathbf {E}}\{N_{t+1}|N_t=N,i_t=i\}= & {} N-r+\frac{i}{N}(q+r), \end{aligned}$$

10 $$\begin{aligned} {\mathbf {E}}\{i_{t+1}|N_t=N,i_t=i\}= & {} i+p_{\text {reproduction},a,t}-i p_{\text {death},a,t}. \end{aligned}$$

**Figure 4:**
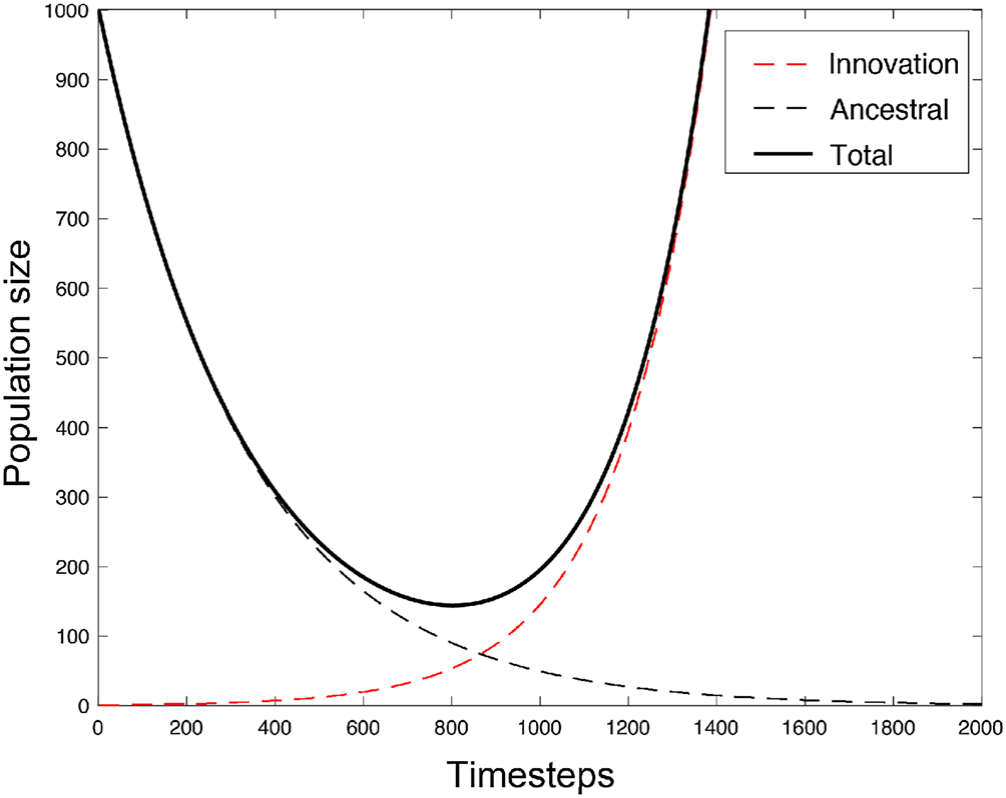
An evolutionary rescue scenario where the ancestral variant causes excess mortality and the novel variant provides a survival benefit. $r=0.003,q=0.005, g=1, f=1, N_0=1000, i_0=1$.

Figure 4 illustrates Eqs. (9) and (10) with unbiased cultural transmission. It is clear that the population size declines sharply after the environmental shift at $T_0=0$ (see black solid line) but the population is rescued through the increasing number of individuals having adopted variant *a* (see red dashed line).

**Figure 5:**
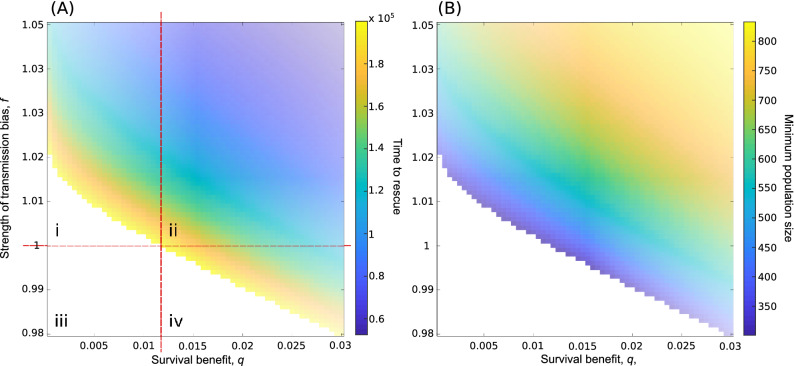
(**A**) The mean time to evolutionary rescue and (**B**) the severity of the population bottleneck. $r=0.0004, g=1, N_0=1000, i_0=1$. Simulations ran until the population was extinct or exceeded its original size, indicating a cultural evolutionary rescue in this parameter range.

Figure 5A shows the interplay between the survival benefit *q* and the cultural transmission advantage *f* of the innovation *a* on the mean time to population rescue, i.e on the mean time until the population reaches its initial size again after a decline. Parameter constellations shown in white do not result in a rescue. We observe a non-linear relationship between *q* and *f*: small values of *q* can be compensated for by large values of *f* (and vice versa) leading to a rapid rescue. Figure 5A shows four parameter domains. The domain labelled (i) contains the cases where cultural transmission bias makes a rescue possible by speeding up the spread of a weakly beneficial trait, where otherwise the population would collapse. The domain labelled (iv) contains the values of *q* for which an evolutionary rescue is possible even in the absence of a transmission advantage to *a*. Finally, domains (ii) and (iii) contain cases where rescues are always possible or never possible (and extinction is likely). Figure 5B shows the corresponding population bottlenecks, i.e. the smallest populations size in the adaptation process. As expected, parameter constellations leading to a long rescue time also lead to small bottlenecks.

We can show that where the mismatch between the environment and the ancestral variant *A* is more pronounced, a rescue is less likely and where they do occur, the population bottleneck is more severe, as one might expect from previous work on evolutionary rescues^11,18,21^.

**Figure 6:**
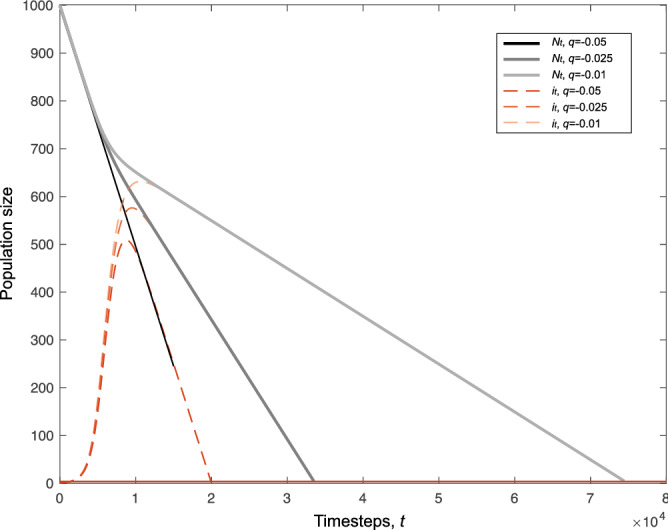
The time to population collapse, where cultural rescues do not occur, for different values of *q*. Threshold for extinction here is 5 individuals and is marked with a red solid line. Black and grey lines show total population sizes, red dashed lines show the frequency of the beneficial cultural trait. Higher frequencies of the beneficial trait delay population collapse—sometimes substantially. $r=0.05, f=2, g=1, N_0=1000$, initial frequency of derived cultural trait is 1/*N*, $i_0=1$.

Finally, we consider the cases in which population extinctions do occur despite the introduction of a beneficial cultural variant. Here, we can show that a weakly beneficial variant, may, sometimes significantly, lengthen the time between the environmental change and population collapse (see Fig. 6). In the absence of a cultural trait, in this system the population will go extinct on average in $\lfloor N_0/r \rfloor$ timesteps. In the presence of a beneficial cultural variant, this time to extinction can be increased considerably. The increase depends on the probability of spread of the cultural variant as well as the protection against death, quantified by *q*, that it confers compared to variant *A*. This lends credence to the idea that the spread of a weakly beneficial cultural trait may facilitate true evolutionary rescue for human populations by prolonging the time a population can wait for a beneficial genetic mutation to arise and spread.

In summary, culture alone may be able to rescue a population, prevent or mitigate population bottlenecks, or extend the survival time of a declining population during which true evolutionary rescue may be possible in a way rare (or unique) for large, long-living organisms.

## Discussion

The uniquely well-developed cognitive and cultural abilities of humans have undoubtedly contributed to our success as a species. However, we understand little about the ways in which our ability to generate and maintain culture affect our adaptation to new environments and our probability of success in those environments. Here we begin to examine these questions in detail by investigating some fundamental questions of cultural adaptation: ‘do we adapt from pre-existing cultural variation or from novel innovation?’, ‘what effects do transmission modes have on this process?’, and ‘how does this process drive successful migration or survival in times of environmental stress?’. We describe a model of the cultural adaptive process, examining aspects of adaptation that are unique to culture, and discuss the possible effects of cultural adaptation on population dynamics.

We begin with a simple system that includes a single finite population in which generations overlap—a condition necessary for understanding the effect of various transmission mechanisms. We focus on a single cultural trait with two possible variants: *A*, an ancestral variant and *a*, a new innovation. We assume that neither variant provides an adaptive benefit before time $T_0$ when an environmental shift occurs. After the shift, however, variant *a* provides a higher benefit.

### Adaptation from standing variation vs. adaptation from de novo innovation

To understand the balance between pre-existing or ‘standing’ cultural variation and de novo innovation in the process of adaptation to a novel environment, we examine the probability of selective sweeps from both sources of variation. To make the links with the genetic literature clear, we have borrowed the term ‘standing variation’ throughout, however in a cultural context we might more often label this ‘cultural memory’. In contrast to the genetic case, for a cultural system it is sensible to assume that innovations are plentiful. Therefore, we assume that there is always one new innovation present in the population prior to an environmental shift, i.e. standing cultural variation exists, and there is little or no ‘waiting time’ before a beneficial innovation arises after an environmental change.

Under these conditions, we show that if the benefit of variant *a*, the variant under selection, is the same in both cases, a selective sweep is more likely from standing variation than from a novel innovation arising after the environmental shift. This is an intuitive consequence of the possibility of variant *a* existing at a frequency higher than 1/*N* at the time of the shift. The frequency dynamic of *a* before the shift, i.e. in the phase where both variants *a* and *A* provide the same level of benefit, can be governed by a variety of distinct transmission mechanisms^5,9^. Here we focus on three transmission mechanisms: unbiased transmission, conformist transmission and anti-conformist transmission. We show that the frequency at which variant *a* is likely to be represented at the time of the environmental shift will change, perhaps dramatically, from one transmission mechanism to another. These changes mean that in the mechanisms we tested the probability of a sweep from standing variation is most likely in the case of anti-conformist transmission where variants are more often represented at intermediate frequencies in the period prior to an environmental change. This suggests that the dominant transmission mechanisms on which a population relies could have a significant effect on its ability to build and maintain cultural diversity and to use that diversity in the case of environmental shock^27^. This echoes the findings of Ref.^28^ who showed that the dominant mode of transmission (in this case horizontal or vertical transmission) could change both the kind of information that spreads in a population and, in turn, the population’s demographic outcomes.

Above, as in a genetic case, innovations are generally assumed to be blind to the exact nature of the environmental shift. Of course, cultural innovations may not be so blind. Humans possess a number of sophisticated cognitive abilities that enable us to generate adaptive culture. We consider two. First, traits may be innovated and deliberately maintained in a population even when the trait is not under selection and the benefit is not immediately clear because we consider it likely to be useful in the future. This ability for foresight means that after an environmental shift a population may possess, in its standing variation, a trait that has higher benefit than we would expect otherwise. Second, although cultural innovation is often modelled as a process of blind variation and selective retention (in analogy to genetic mutation) we may be, in fact, capable of biasing the distribution of benefits of de novo innovations to solve particular environmental ‘problems’. Again, the ability for such ‘directed innovation’ means that when individuals innovate in response to environmental pressure innovation might be more useful or adaptive than we would otherwise expect. We posit that foresight is unlikely to produce a variant that is more beneficial in the novel circumstances than directed innovation. For this reason the existence of these cognitive mechanisms, foresight and ‘directed innovation’, introduces a trade-off and the probability of a selective sweep from standing variation with foresight and de novo directed innovation is no longer intuitive. The model shows that where foresight is poor and directed innovations are relatively good, novel innovations in response to environmental shifts are more likely to produce a cultural selective sweep. In the case where foresight is relatively good (or, alternatively, the environmental shift is predictable to some extent) a sweep from standing variation is more likely. This is true even when innovation after the shift can produce a cultural trait with a larger benefit.

The resulting shift in the balance between initially neutral traits maintained at a frequency greater than 1/*N* and the potential selective advantage of newly innovated traits raises important questions about how standing variation is produced and maintained. In cultural systems where foresight and directed innovation are absent^29^ (and indeed in genetic systems where the same is true^30^), a rapidly fluctuating environment generally favours an increase in innovation rate. However, when foresight is included, a variety of strategies for maintaining useful standing variation may be favoured instead. For example, selectively maintaining older information, neutral or even currently maladaptive traits, or switching reliance on transmission mechanisms to maintain more information that may be useful in the future.

### Evolutionary rescue

One of the most important effects of environmental shifts, both in terms of migration and environmental changes, might be the effect on a population’s demography and survival probability. Our understanding of the interactions between how culture is maintained by a population, the environment and, for example, population size is incomplete (and the subject of some controversy e.g.^31–34^). Our understanding of the effect of population bottlenecks in a cultural system is even more limited but see Ref.^35^. Here we attempt to scratch the surface of these issues. We described a model of ‘cultural rescue’ where a population undergoes a large and catastrophic environmental change leading to population decline. The decline may be halted by the spread of a cultural trait that can increase an individual’s survival probability. We show that the survival benefit of the trait itself can lead to a population rescue under unbiased transmission. Further, including the effect of biasing transmission towards the beneficial variant can reduce the time to such a rescue. Cultural traits can produce a rescue where one otherwise would not occur. Finally, and importantly, a beneficial cultural trait can reduce the severity of a population bottleneck. This raises the possibility that a rapid cultural response to a massive environmental shift might maintain the population at higher numbers for longer, enabling a (slower) evolutionary rescue where one might not otherwise occur. Our results suggest that a gene-culture co-evolutionary model of evolutionary rescue would be more appropriate and indeed may be necessary to fully understand human adaptation specifically.

The widening of a population bottleneck by behavioural or cultural adaptation might also have an important effect on our ability to detect such culturally buffered selective events in genetic data. In fact, it may be that such events could only be detected in cultural data and, at that, for a very short time.

As humans moved across the planet, colonising almost every continent and establishing themselves as a hugely successful species from a demographic point of view, populations inevitably experienced many large-scale environmental changes and shifts—from food scarcity to large average temperature differences—from one location to another. To understand how human populations successfully navigated, and continue to navigate, these challenges we must understand more than just the process of genetic adaptation. Drawing on models like the ones above and existing theory of cultural evolution, we must generate a much deeper understanding of the mechanics of cultural adaptation and its inevitable interactions with our genes.

## Supplementary information

Supplementary Information.
