# Supplementary material for: The fundamentals of cultural adaptation: implications for human adaptation
Source: Sci Rep. 2020 Aug 31;10:14318. doi: 10.1038/s41598-020-70475-3 (PMC7459347; doi:10.1038/s41598-020-70475-3)
Supplement: Supplementary file 1 — Supplementary Information. [file 41598_2020_70475_MOESM1_ESM.pdf]

The fundamentals of cultural adaptation: implications for human adaptation (Supplementary Material)

Laurel Fogarty[1,*] and Anne Kandler[1,+]

[1]Theory in Cultural Evolution Lab, Department of Human Behavior, Ecology and Culture, Max Planck Institute for Evolutionary Anthropology
*laurel_fogarty@eva.mpg.de
+anne_kandler@eva.mpg.de

August 10, 2020

All derivations shown in sections S1 to S3 are well-known [see e.g. 1].

S1 The probability of fixation from *de novo* innovation

We consider a situation where a variant A has gone to fixation in a population of size N. At time T_0 an environmental change occurs and immediately afterwards a novel variant a appears with frequency $1/N$. We assume that now variant a provides an adaptive benefit f and variant A provides a benefit g (with $f > g$ and w.l.o.g. $g = 1$) to their adopters. In the following we calculate the probability that a novel variant a will go to fixation under payoff-biased transmission. In this case the transition probabilities of the Markov process X_t are given by

$$\begin{aligned} p_{i,i-1} &= \frac{g(N-i)}{fi+g(N-i)}\frac{i}{N} = \beta_i \\ p_{i,i+1} &= \frac{fi}{fi+g(N-i)}\frac{N-i}{N} = \alpha_i \\ p_{i,i} &= 1 - P_{i,i+1} - P_{i,i-1} = 1 - \alpha_i - \beta_i. \end{aligned}$$

Using these transition probabilities, the following recursion relation for π_i, the fixation probability from an initial frequency i/N, can be defined

$$\pi_i = \beta_i \pi_{i-1} + (1 - \alpha_i - \beta_i)\pi_i + \alpha_i \pi_{i+1}.$$

Rearranging leads to

$$\beta_i(\pi_i - \pi_{i-1}) = \alpha_i(\pi_{i+1} - \pi_i)$$

and with $y_i = \pi_i - \pi_{i-1}$ and setting $\gamma_i = \frac{\beta_i}{\alpha_i}$ we obtain

$$y_{i+1} = \gamma_i y_i.$$

Further, it holds $\sum_{i=1}^m y_i = \pi_m$ and $y_k = \pi_1 \prod_{l=1}^{k-1} \gamma_l$. Because

$$\pi_i = y_1 + y_2 + y_3 ... y_i$$

$$\pi_i = \pi_1 + \pi_1 \prod_{k=1}^{2-1} \gamma_k + \pi_1 \prod_{k=1}^{3-1} \gamma_k ... \pi_1 \prod_{k=1}^{i-1} \gamma_k$$

we get that

$$\pi_i = \pi_1 + \pi_1 \sum_{l=1}^{i-1} \prod_{k=1}^{l} \gamma_k.$$

Using the boundary condition $\pi_N = 1$, we obtain

$$\pi_1 + \pi_1 \sum_{l=1}^{N-1} \prod_{k=1}^{l} \gamma_k = 1.$$

This finally gives an expression for the probability of fixation from a *de novo* innovation with adaptive benefit f

$$\pi_{\mathrm{DN}} = \pi_1 = \frac{1}{1 + \sum_{l=1}^{N-1} \prod_{k=1}^{l} \gamma_k}. \tag{S1}$$

S2 The probability of fixation from standing variation

Now we assume that the innovation of variant a occurred some time before the environmental change and unbiased transmission has caused it to reach frequency j/N, with $j = 1, \dots, N-1$ at T_0. We condition on the existence of an innovation that has not yet reached fixation in the population. Consequently, the probability of fixation of variant a after the environmental change will depend not just on the adaptive benefit of a but also on the frequency of a. To account for that we first calculate the probability that variant a has frequency j/N under unbiased transmission and multiply this by the probability of fixation from frequency j.

In the case of unbiased transmission, the transition probabilities of the Markov process X_t described above are

$$p_{i,i-1} = p_{i,i+1} = \frac{i(N-i)}{N^2} = a_i \quad \text{and} \quad p_{i,i+1} = \frac{i^2 + (N-i)^2}{N^2} = 1 - 2a_i.$$

It holds

$$\mathbf{E}\{X_t | X_{t-1} = i\} = (i-1)a_i + i(1-2a_i) + (i+1)a_i = i$$

and applying the law of iterated expectation leads to

$$\mathbf{E}\{X_t | X_0 = i\} = i. \tag{S2}$$

Let τ be the stopping time, i.e. the time until the process X_t reaches either 0 or N, the two absorbing boundaries of the considered Markov process. Using Eq. (S2) we obtain

$$\mathbf{E}\{X_\tau | X_0\} = NP(X_\tau = N | X_0 = i) + 0P(X_\tau = 0 | X_0 = i) = i$$

which determines the fixation probability

$$P(X_\tau = N | X_0 = i) = \frac{i}{N}.$$

We assume in the following that $X_0 = i$, ie. the Markov process starts in state i. To calculate the time to absorption, t_i, we first calculate the mean times, t_{ij}, that X_t is in state j before absorption. It holds

$$t_{ij} = a_i t_{i-1,j} + (1-2a_i) t_{ij} + a_i t_{i+1,j} + \delta_{ij} \tag{S3}$$

where δ_{ij} is the Kronecker symbol. Eq. (S3) simplifies to a difference equation of second order

$$t_{i+1,j} - 2t_{ij} + t_{i-1,j} = -\frac{\delta_{ij}}{a_i}$$

with the boundary conditions $t_{0j} = 0$ and $t_{Nj} = 0$. To solve this equation we define $x_i = t_{ij} - t_{i-1,j}$ and obtain

$$x_{i+1} = x_i - \frac{\delta_{ij}}{a_i}.$$

This leads to the recursions

$$\begin{aligned} x_1 &= t_{1j} - t_{0j} = t_{1j}, \\ x_2 &= x_1 - \frac{\delta_{1j}}{a_1}, \\ x_3 &= x_1 - \frac{\delta_{1j}}{a_1} - \frac{\delta_{2j}}{a_2}, \\ &\vdots \\ x_s &= x_1 - \sum_{i=1}^{s-1} \frac{\delta_{ij}}{a_i} = \begin{cases} x_1 & j \geq s \\ x_1 - \frac{1}{a_j} & j < s \end{cases} = \begin{cases} t_{1j} & j \geq s \\ t_{1j} - \frac{1}{a_j} & j < s. \end{cases} \end{aligned}$$

It further holds that

$$t_{sj} = \sum_{i=1}^{s} x_i = \begin{cases} st_{1j} & j > s \\ st_{1j} - \frac{s-j}{a_j} & j \leq s. \end{cases}$$

To calculate t_{1j} we use the boundary condition $t_{Nj} = 0$. We have

$$t_{Nj} = \sum_{i=1}^{N} x_i = Nt_{1j} - \frac{N-j}{a_j} = 0$$

and therefore

$$t_{1j} = \frac{N-j}{Na_j} = \frac{N-j}{N\frac{j(N-j)}{N^2}} = \frac{N}{j}.$$

Summarising, we obtain

$$t_{sj} = \begin{cases} N\frac{s}{j} & j > s \\ N\frac{s}{j} - \frac{s-j}{\frac{j(N-j)}{N^2}} = \frac{N(N-s)}{N-j} & j \leq s \end{cases}$$

which lastly leads to

$$t_s = \sum_{j=1}^{N-1} t_{sj} = \sum_{j=1}^{s} t_{sj} + \sum_{j=s+1}^{N-1} t_{sj} = N(N-s) \sum_{j=1}^{s} \frac{1}{N-j} + Ns \sum_{j=s+1}^{N-1} \frac{1}{j}.$$

For a starting frequency of $1/N$, the time spent in any state j is given by

$$t_{1j} = \frac{N}{j} \tag{S4}$$

and the total time such a variant exists before absorption is

$$t_1 = N + N \sum_{k=2}^{N-1} \frac{1}{k}. \tag{S5}$$

Consequently, the probability that variant a possesses frequency j is determined by

$$\frac{t_{1j}}{t_1} = \frac{1}{j\left(1 + \sum\limits_{k=2}^{N-1} \frac{1}{k}\right)}.$$

Lastly, we generalise the expression for the fixation probability (S1) from a starting frequency of $1/N$ to a general starting frequency of j/N using the fact that

$$\pi_j = \pi_1 + \pi_1 \sum_{l=1}^{j-1} \prod_{k=1}^{l} \gamma_k.$$

Using

$$\pi_1 = \frac{1}{1 + \sum\limits_{l=1}^{N-1} \prod\limits_{k=1}^{l} \gamma_k}.$$

we obtain

$$\pi_j = \frac{1 + \sum\limits_{l=1}^{j-1} \prod\limits_{k=1}^{l} \gamma_k}{1 + \sum\limits_{l=1}^{N-1} \prod\limits_{k=1}^{l} \gamma_k}. \tag{S6}$$

Summarising, the probability of a fixation from standing cultural variation, at the time of an environmental change, i.e. from a variant with frequency j/N at T_0 and an adaptive benefit f, is given by

$$\pi_{\text{SV}} = \sum_{j=1}^{N-1} \frac{t_{1j}}{t_1} \cdot \pi_j \tag{S7}$$

with t_{1j}, t_1 and π_j defined by Eqs. (S4), (S5) and (S6), respectively.

S3 The probability of fixation from standing variation under alternative transmission mechanisms

Different cultural transmission processes change the way cultural variants are maintained or lost in a population, in particular they change the frequency distribution of the variants in the population. As a result the probability of a sweep to fixation from standing cultural variation may depend on the cultural transmission process present in the population before T_0. To quantify the effects of different transmission processes, we calculate the probability that variant a has frequency j conditioned on an arbitrary transmission process expressed by the transition probabilities $p_{i,i-1} = \beta_i, p_{i,i+1} = \alpha_i$, and $p_{i,i} = 1 - \alpha_i - \beta_i$. To do so we have to generalise the calculations of the times to absorption, t_1, (S5) and time at a given frequency, t_{1j}, (S4) to allow for such general transition probabilities (see Eq. (S7)).

Reformulating Eq. (S3), we obtain

$$t_{ij} = \beta_i t_{i-1,j} + (1 - \alpha_i - \beta_i) t_{ij} + \alpha_i t_{i+1,j} + \delta_{ij}.$$

Simplifying and defining $\gamma_i = \frac{\beta_i}{\alpha_i}$ and $x_i = t_{ij} - t_{i-1,j}$ leads to

$$x_{i+1} = \gamma_i x_i - \delta_{ij}.$$

The recursion equations for x_s with $1 < s < N - 1$ have then the form

$$x_s = t_{1j} \prod_{l=1}^{s-1} \gamma_l - \sum_{l=1}^{s-1} \prod_{k=l+1}^{s-1} \gamma_k \frac{\delta_{lj}}{\alpha_l}.$$

Using the boundary condition $t_{Nj} = 0$ we obtain the expression for t_{1j}

$$t_{1j} = \frac{\frac{(N-j)}{\alpha_j} \prod_{k=j+1}^{N-1} \gamma_k}{N \prod_{l=1}^{N-1} \gamma_l}.$$

which allows us to calculate the time to absorption

$$t_1 = \sum_{j=1}^{N-1} t_{1,j}.$$

S4 'Trait frequency spectrum' for an infinite site Moran model

Knowing the ratio of t_{1j}, the mean time that the Markov process X_t with the initial condition $X_0 = 1$ was in state j, to t_1, the mean time that variant a exists before absorption into either state 0 or N, allows us to derive a 'trait frequency spectrum' for an infinite sites Moran model under the transmission process defined by the general transition probabilities α_i and β_i [1]. For conformity and anti-conformity the expressions for α and β are given by

$$p_{i,i+1} = \frac{(i/N)^{(1+\theta)}}{(i/N)^{(1+\theta)} + (1-(i/N))^{(1+\theta)}} \frac{N-i}{N} = \alpha_i, \quad \text{(S8)}$$

$$p_{i,i-1} = \frac{((N-i)/N)^{(1+\theta)}}{(i/N)^{(1+\theta)} + (1-(i/N))^{(1+\theta)}} \frac{i}{N} = \beta_i, \quad \text{(S9)}$$

We generate an expression for the average number of cultural variants expected to be present in the population at some timestep t, denoted by S_N. If t_1 is the number of time steps for which a variant with a starting frequency of $1/N$ persists in the population, the probability of losing a variant in one generation, i.e. in N timesteps, is given by N/t_1. Therefore, the number of variants expected to be lost in one generation is given by $(NS_N)/t_1$. In contrast, the number of variants innovated per generation is given by $N\mu$ where μ is the *per capita* innovation rate. At equilibrium, the number of variants gained and lost must be equal leading to the expression

$$\frac{NS_N}{t_1} = N\mu,$$

and consequently

$$S_N = t_1\mu.$$

We know that the probability that a variant has frequency j/N is given by $\frac{t_{1j}}{t_1}$ and therefore we can derive the number of variants with frequency j in the population, denoted by $S_{N,j}$, as

$$S_{N,j} = \frac{t_{1j}}{t_1} S_N = t_{1j}\mu.$$

Fig. S1 shows the 'trait frequency spectra' for all transmission processes considered. While conformity results in a situation where most variants possess a very low frequency (see black solid line) the opposite is true for anti-conformity (see black dash-dot line); here many variants remain at intermediate frequencies for a relatively long period of time. Unbiased transmission (see dashed line) is between conformity and anti-conformity but without being skewed to the right.

References

[1] Warren John Ewens. *Mathematical Population Genetics: Theoretical introduction*, volume 27. University Of Chicago Press, 2004.

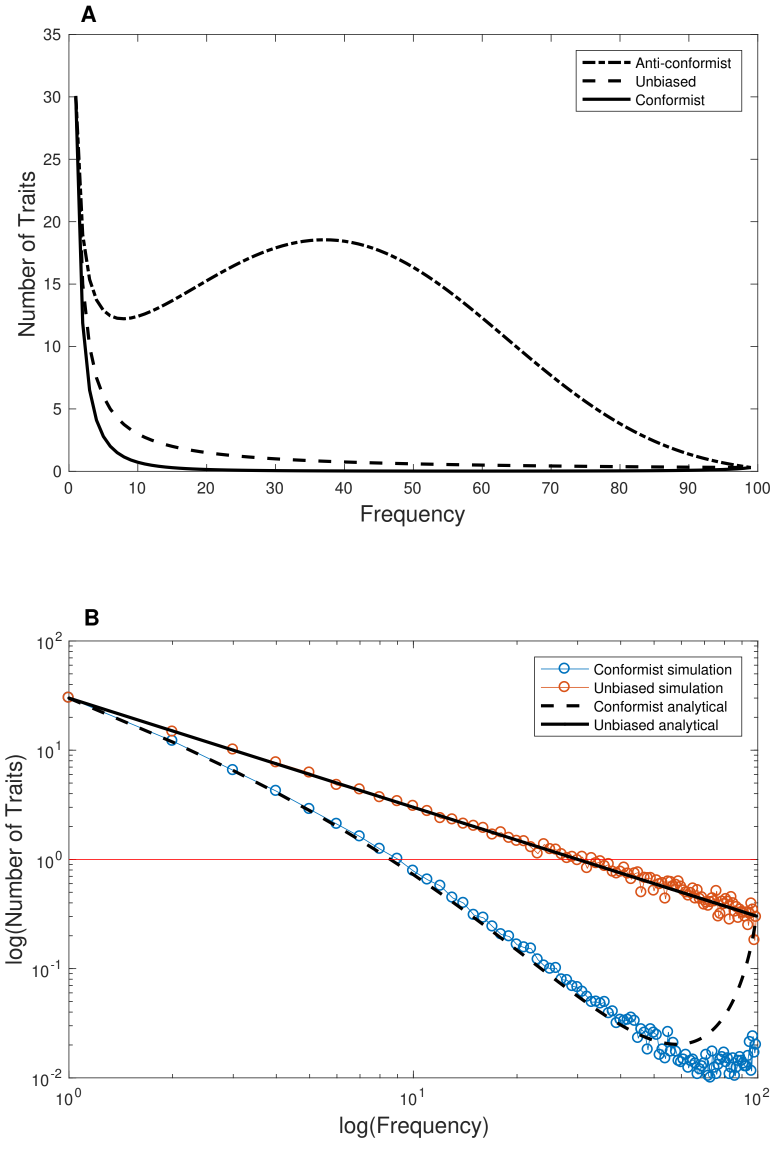

Figure S1: (A) Trait frequency spectrum under unbiased transmission (dashed line), conformity with $\theta = 0.05$ (black solid line), anti-conformity with $\theta = -0.05$ (black dot-dash line) and $N = 100$, $\mu = 0.3$. (B) Mean simulation results for the same system with $N = 100$, $\mu = 0.3$, number of timesteps $= N(500)$, 2000 simulation repeats. Note log-log plot to better demonstrate the difference between transmission mechanisms in the tails.
